# Gut microbiota and metabolic status during pregnancy in captive Asian elephants

**DOI:** 10.3389/fvets.2026.1749490

**Published:** 2026-03-09

**Authors:** Fanwen Zeng, Tingting Zhu, Xuanjiao Chen, Kang Huang, Lu Liu, Guoqian Wang, Jiewen Mai, Shouquan Zhang

**Affiliations:** 1 Guangzhou Zoo and Guangzhou Wildlife Research Center, Guangzhou, China; 2 Microbiome Research Group, Research Center for Life Science and Healthcare, Nottingham Ningbo China Beacons of Excellence Research and Innovation Institute (CBI), University of Nottingham Ningbo China, Ningbo, China; 3 National Engineering Research Center for Breeding Swine Industry, Guangdong Provincial Key Lab of Agro-Animal Genomics and Molecular Breeding, College of Animal Science of South China Agricultural University, Guangzhou, China

**Keywords:** Asian elephants (*Elephas maximus*), captive, gut microbiota, metabolome, pregnant

## Abstract

**Background:**

The gut microbiota is regarded as one of the key factors regulating host health. The gut microbiota and its connection to fecal metabolites are crucial for supporting fetal development and ensuring maternal health during reproductive stages. Although studies have examined Asian elephants, the composition and function of the gut microbiota in pregnant and non-pregnant captive Asian elephants have not been reported.

**Methods:**

We compared the fecal microbiota and fecal metabolites of pregnant (G1), non-pregnant (never gotten pregnant after reaching sexual maturity, G2), and subadult (G3) captive Asian elephants using metagenomic sequencing and untargeted liquid chromatography-tandem mass spectrometry (LC-MS/MS) metabolomics.

**Results:**

We found significant differences in the gut microbiota among the G1, G2, and G3 groups. The phylum *Bacteroidetes* showed notable differences between G1 and G2. The analysis of fecal metabolomics revealed significant differences in 49 metabolites between G1 and G2, of which 25 were upregulated and 24 were downregulated. These results suggested significant differences in the composition of gut microbiota and fecal metabolites during reproductive stages, while gut microbial diversity remained stable. These findings inform our ongoing research on the potential health conditions of captive Asian elephants, with the aim of better understanding the role of the gut microbiota in reproductive regulation.

## 1 Introduction

The formation and proliferation of the gut microbiota begin at the time of an animal's birth, and their composition and function are primarily determined by various environmental, nutritional, and genetic factors (1, 2). Microbiota acquired at birth develops in parallel with the host and maintains temporal stability and diversity throughout adulthood until death, all within a dynamic equilibrium (3, 4). The three main stages in female animals are growth, adulthood, and pregnancy. Changes in these physiological stages affect the energy and nutritional requirements of females (5). Compared with the cycling state, an animal's daily energy intake increases by 20%−30% during pregnancy (6). Changes also occur in maternal hormones, immunity, and metabolism, leading to alterations in the diversity and structure of the gut microbiota during pregnancy (7, 8). An increasing number of researchers have reported emerging information on host-microbe interactions during pregnancy, revealing a strong correlation between intestinal microbiota and animal reproductive success (9). The gut microbiota and their metabolites regulate various activities in the placenta and uterus, thereby influencing pregnancy outcomes.

The Asian elephant (*Elephas maximus*) is the largest terrestrial mammal in Asia. It is listed as an endangered species by the International Union for Conservation of Nature (IUCN) (10), and captive breeding is one of the strategies employed to prevent its extinction (11, 12). However, many captive Asian elephants have difficulty maintaining their population size, exhibiting significantly lower reproductive efficiency compared with wild Asian elephants (13). The reproductive performance of captive Asian elephants may be affected by several factors, including their health status and feeding management. Free ranging elephants enjoy freedom of selection of food items and they may select forages supplying nutrients according to their requirements (14). Subadult elephants and pregnant elephants should consume more energy and protein-rich feed than adult elephants (15, 16). In addition, females undergo complex physiological and biological changes during growth, adulthood, and pregnancy, which are crucial for the health of both the mother and the fetus. Currently, the composition and function of the female gut microbiota and its metabolites in regulating physiological and metabolic processes during the different life stages of captive Asian elephants remain unclear.

In the present study, metagenomic sequencing and untargeted liquid chromatography—tandem mass spectrometry (LC—MS/MS) metabolomics were performed on fecal samples from three pregnant captive Asian elephants (G1), three non-pregnant captive Asian elephants (never gotten pregnant after reaching sexual maturity, G2), and three subadult captive Asian elephants (G3), in order to explore differences in their gut microbiota and the related metabolic pathways. Based on this, we aimed to gain a better understanding the differences in the gut microbiota and endogenous metabolites in G1, G2, and G3. Provide references for implementing appropriate diet and scientific management for the captive Asian elephants.

## 2 Materials and methods

### 2.1 Sample collection

All animal procedures were conducted by the guidelines for the review of laboratory animal welfare and ethics in Guangdong Province. All animal procedures were approved by the Animal Care and Use Committee of Guangzhou (#YL202401) and Gongguan Zoo. All fecal samples were collected during the spring months (March to April 2024) at Dongguan Zoo in Guangdong Province, China, from 10 Asian elephants (*Elephas maximus*) aged 9–37 years, including pregnant adults (G1, *n* = 3), non-pregnant adults (G2, *n* = 3), and subadult elephants (G3, *n* = 4). Since March all elephants have been on the same diet which included oat grass (*Arrhenatherum elatius*), Napier grass (*Pennisetum purpureum Schumach*), corn flour, soybean meal, alfalfa, apples, bananas, carrots, and pumpkin twice daily, and were provided with mixed hay and water *ad libitum*. Fecal samples were collected from the ground within 2 h after defecation and before morning feeding, and transported to the laboratory at a low temperature within 4 h. Upon arrival, each sample was divided into two portions and stored at −80 °C until analysis. One portion was used for metagenomic sequencing analysis, and the other portion was used for metabolomic analysis. Detailed information on captive Asian elephants is listed in Table 1.

**Table 1:** Basic information of the elephants included in the study.

**Group**	**Name**	**Age (year)**	**Reproductive state**
G1	A1	37	Pregnant
	A2	24	Pregnant
	A3	31	Pregnant
G2	A4	28	Non pregnant
	A5	35	Non pregnant
	A6	42	Non pregnant
G3	A7	5	Non pregnant
	A8	4	Non pregnant
	A9	6	Non pregnant
	A10	11	Non pregnant

### 2.2 DNA Extraction and sample testing

Fecal DNA was extracted using the QIAamp PowerFecal Pro DNA Kit (Qiagen, Düsseldorf, North Rhine-Westphalia, Germany) according to the manufacturer's instructions. DNA degradation and potential contamination were assessed using 1% agarose gel electrophoresis. DNA concentration was measured with a Nanodrop spectrophotometer (Thermo Fisher Scientific, Waltham, MA, United States) and adjusted to 1 ng/μL. Samples with OD values between 1.8 and 2.0 were selected for sequencing.

### 2.3 Metagenomic sequencing and bioinformatics analysis

One microgram of DNA per sample was used as the sequencing template. Sequencing libraries were prepared using the NEBNext^®^ Ultra™ DNA Library Prep Kit for Illumina (NEB, Ipswich, MA, United States), following the manufacturer's instructions. Briefly, DNA samples were fragmented to approximately 350 bp using a Covaris ultrasonic disruptor (Woburn, MA, United States). The DNA fragments were subjected to end polishing, A-tailing, adapter ligation, purification, and PCR amplification to complete library preparation. Next, the sample was preliminarily quantified using Qubit 2.0 (Thermo Fisher Scientific, Waltham, MA, United States) and diluted to 2 ng/μL. The insert size of the library was assessed using an Agilent 2100 bioanalyzer (Agilent Technologies, California, United States). The effective concentrations of the samples were accurately quantified using real-time PCR (effective concentration > 3 nM). Based on the effective library concentration and required data output, the libraries were sequenced on an Illumina NovaSeq 6000 (Illumina, California, United States), generating 150 bp paired-end reads.

Raw data obtained from the NovaSeq sequencing platform were preprocessed using fastp software (default parameters, https://github.com/OpenGene/fastp) to generate clean data for subsequent analysis. Adapter sequences, sequences shorter than 50 base pairs, and low-quality sequences (default threshold ≤ 20) were removed from the raw data. The processing steps were as follows: (1) Paired reads were removed if either read contained adapter sequences; (2) Paired reads were removed if the number of low-quality bases (Q ≤ 5) in either read exceeded 50% of that read's total bases; (3) Paired reads were removed if the *N* content in either read exceeded 10% of that read's total bases. To eliminate potential host contamination, clean reads were aligned to the host genome, and potential host reads were removed by mapping all clean reads against the host genome using Bowtie2 version 2.2.4 (http://bowtie-bio.sourceforge.net/bowtie2/index.shtml; end-to-end, very-sensitive) (17). Read pairs for which either mate aligned to the host genome were discarded, and only the remaining non-host reads were used for downstream assembly, gene prediction, taxonomic profiling, and functional annotation.

MEGAHIT software (https://www.metagenomics.wiki/tools/assembly/megahit) was used to assemble and analyze clean data from fecal samples. Scaffolds containing ambiguous *N* bases were split to obtain scaffolds without any “*N*” components. Fragments shorter than 500 bp were filtered from scaftigs generated by single-sample assembly, followed by statistical analysis and gene prediction.

MetaGeneMark (http://topaz.gatech.edu/GeneMark/) was used to predict open reading frames (ORFs) for scaftigs ≥500 bp in each sample (18). Using the default parameters, predicted sequences shorter than 100 nucleotides were filtered out (19). To remove redundancy from the ORF prediction results, CD-HIT software (V4.5.8, http://www.bioinformatics.org/cd-hit/) was used to cluster gene sequences with 90% identity and 90% coverage, generating a non-redundant initial gene catalog (20). Clean data from each sample were aligned to the initial gene catalog using Bowtie2 version 2.2.4, and the number of reads matching each gene was calculated. Genes with two or fewer reads in each sample were filtered out to obtain the final gene catalog (unigenes) for subsequent analysis. The abundance of each gene in each sample was calculated based on the number of aligned reads and gene length, using a specified formula. Basic statistical analyses were performed using the abundance data for each gene across samples.

Amino acid sequences were aligned to the Micro_NR database using DIAMOND software (Drost, Tübingen, Germany). (V0.9.9, https://github.com/bbuch fink/diamond/) for species annotation (parameter settings: blastp, e-value is 1e−5) (21, 22). LCA analysis (https://en.wikipedia.org/wiki/Lowest_common_ancestor) was conducted to determine the species annotation of this sequence (23). Based on the LCA annotation results and gene abundance, the abundance information and number of genes in each sample at each classification level (kingdom, phylum, class, order, family, genus, and species) were obtained (17). Based on the abundance at each classification level, Krona analysis, relative abundance profiles, and an abundance clustering heatmap were generated (24). Pareto-scaled principal component analysis (PCA, R ade4 package) and non-metric multidimensional scaling (NMDS, R vegan package) were used to perform dimension-reduction analysis. ANOSIM analysis (R vegan package) was conducted to examine differences between the groups. Subsequently, Metagenomeseq and LEfSe analyses (Harvard, Massachusetts, United States), were performed to identify species that differed between the groups. Metastats analysis was used to conduct permutation tests for each taxon. After obtaining the *P*-value, the Benjamini-Hochberg false discovery rate (FDR) was used to obtain the q-value, which was used to correct for multiple comparisons (25). LEfSe analysis was conducted using the LEfSe software (default LDA score set to 4). Finally, a random forest analysis (R pROC and randomForest packages) was applied to select species at the genus level in a gradient manner and to construct a random forest model. Significant species were selected based on the mean decrease in accuracy and the mean decrease in gin. Cross-validation was then performed (default 10-fold) for each model.

DIAMOND software (https://github.com/bbuchfink/diamond/) was used to align unigenes against several functional databases, including KEGG (http://www.kegg.jp/kegg/), eggNOG (http://eggnogdb.embl.de/#/app/home), and CAZy (http://cazy.org/). For each sequence alignment, the sequence with the highest score was selected for subsequent analyses.

### 2.4 Untargeted LC-MS/MS metabolomics

The samples (100 mg) were individually ground using liquid nitrogen. Next, 500 μL of 80% methanol was added to each sample, followed by vortex mixing. The mixture was then incubated in an ice bath for 5 min. After centrifugation at 15,000 × *g* for 20 min at 4 °C, the supernatant was diluted with high-purity water to achieve a final methanol concentration of 53%. The samples were centrifuged again at 15,000 × *g* and 4 °C for 20 min, and the supernatant was collected for LC–MS analysis.

Untargeted high-performance LC-MS/MS analysis was conducted at Novogene Co., Ltd. (Beijing, China) using an ultra-high-performance liquid chromatography (UHPLC) system (Vanquish UHPLC, Thermo Fisher Scientific) coupled with a high-resolution mass spectrometer (Q Exactive™ HF-X, Thermo Fisher). For chromatographic separation, dried metabolite samples were resuspended in a 1:1 (v/v) mixture of 0.1% formic acid in water (mobile phase A) and 0.1% formic acid in acetonitrile (mobile phase B). A 10 μL aliquot of each sample was automatically injected into the UHPLC system and separated on a Hypersil Gold column (C18, Thermo Fisher Scientific) at a flow rate of 0.2 mL/min and a temperature of 40 °C.

Gradient elution was performed using water + 0.1% formic acid (A) and acetonitrile/isopropanol (1:1) (B) as the mobile phases. From 0 to 3 min, the value of A decreased from 98 to 0%, whereas that of B increased from 2 to 85%. During the period of 3 to 10 min, A remained at 0%, whereas B increased from 85 to 100%. During the period from 10.0 to 10.1 min, A increased from 0 to 98%, while B decreased from 100 to 2%. From 10.1 to 12 min, A and B were maintained at 98% and 2%, respectively.

Mass spectrometry data were acquired in both positive (+ESI) and negative (–ESI) electrospray ionization modes, with a scanning range of m/z 100–1,500 Da. The ion spray voltage was 3.5 kV, sheath gas flow rate was 35 psi, and auxiliary gas flow rate was 10 L/min. The capillary temperature was 320 °C, S-lens RF level was 60, and auxiliary gas heater temperature was 350 °C. Polarity was positive and negative. Secondary MS/MS scans were performed using data-dependent acquisition.

Compound Discoverer (version 3.1; Thermo Fisher Scientific, Waltham, MA, United States) was used for peak alignment, peak picking, and quantitative data processing. The molecular formula was predicted based on the molecular ion peak and fragment ions and compared with the mzCloud (https://www.mzcloud.org/), mzVault (Thermo Fisher Scientific), and Masslist databases (local database, Novogene Co., Ltd.). Quality control (QC) samples were processed alongside the biological samples. Compounds with a relative peak area coefficient of variation (CV) greater than 30% in the QC samples were excluded. Finally, the metabolites were identified and quantified. Statistical analyses were performed using the statistical software R (https://www.r-project.org/), Python (https://www.python.org/downloads/release/python-276/), and the Linux operating system (CentOS 6.6, RedHat, Raleigh, NC, United States).

The identified metabolites were annotated using the KEGG (https://www.genome.jp/kegg/pathway.html), HMDB (https://hmdb.ca/metabolites), and LIPIDMAPS (http://www.lipidmaps.org/) databases. After processing the data using the metabolomics software metaX, PCA and partial least squares discriminant analysis (PLS-DA) were performed to obtain the variable importance in projection (VIP) values for each metabolite. In the univariate analysis, *t*-tests were used to assess the statistical significance (*P*-value) of each metabolite between the two groups, and the fold change (FC) of metabolites was calculated. The default criteria for screening differential metabolites (DMs) were VIP > 1, *P*-value < 0.05, and FC ≥ 2 or FC ≤ 0.5. MetaboAnalyst 5.0 (http://www.metaboanalyst.ca) was used to perform clustering and metabolic pathway analyses of selected DMs.

### 2.5 Statistical analysis

Statistical analysis was conducted using the SPSS 22.0 software package and GraphPad Prism 9 (California, United States). The experimental data are expressed as the mean ± SEM. Measurements indexes were compared using one-way ANOVAs and Kruskal–Wallis test with ^*^P < 0.05, ^**^P < 0.01, ^***^P < 0.001.

## 3 Results

### 3.1 Generation and analysis of metagenomic sequencing data

The raw data obtained from Illumina sequencing contained some low-quality reads. To obtain valid sequences for subsequent analysis, the raw data were preprocessed to ensure the accuracy and reliability of the subsequent analysis results. The statistical results of sequencing data preprocessing are shown in Supplementary Data Sheet 2. The Venn diagram shows that 1,712,850 genes were detected across all three groups, with 102,616 genes uniquely detected in G1, 171,720 genes uniquely detected in G2, and 204,703 genes uniquely detected in G3 (Supplementary Data Sheet 1).

### 3.2 Differences in the species and abundance of gut microbiota

**Figure 1:**
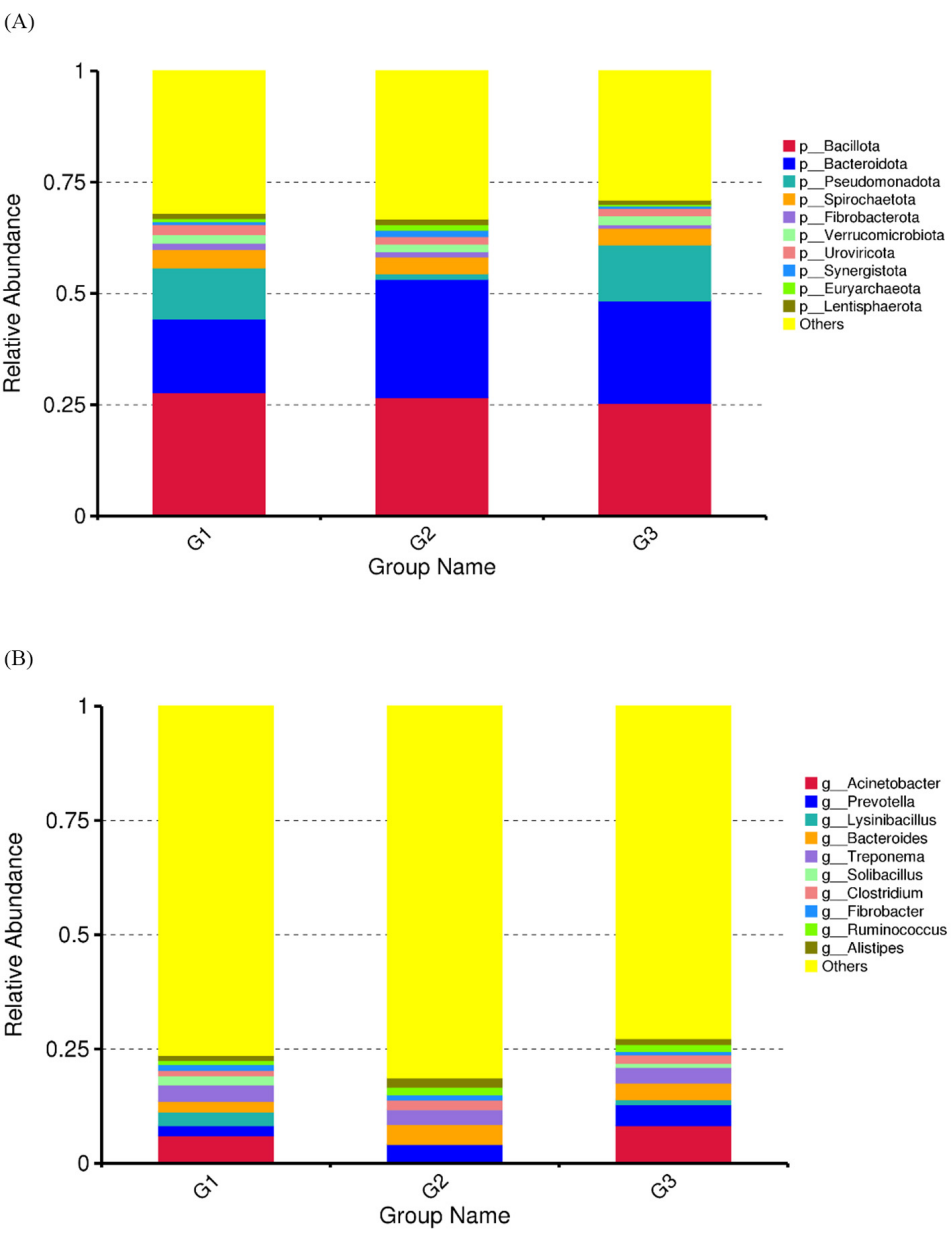
Relative abundance analysis of gut microbiota in G1, G2, and G3. **(A)** The bar plot showing the top 10 gut microbiota of the phylum. **(B)** The bar plot showing the top 10 gut microbiota of the genus. The unannotated category denotes genes/contigs that, after host-read removal, did not meet alignment thresholds for assignment to a known species or functional entry in KEGG/eggNOG; this high proportion reflects limited reference coverage for wildlife gut microbiota rather than residual host contamination.

The relationship between gut microbiota composition and different physiological periods was assessed by comparing changes in relative microbial abundance and diversity across the G1, G2, and G3 groups. The composition of the gut microbiota in the three groups is shown in Figure 1. G1 and G3 showed consistent grouping patterns in several core phyla (e.g., *Bacteroidetes, Actinomycetota*, and *Pseudomonadota*); however, G2 showed different phylogenetic enrichment/deficiency characteristics compared to G1 and G3 (Figures 1A, B, Supplementary Data Sheet 3).

**Figure 2:**
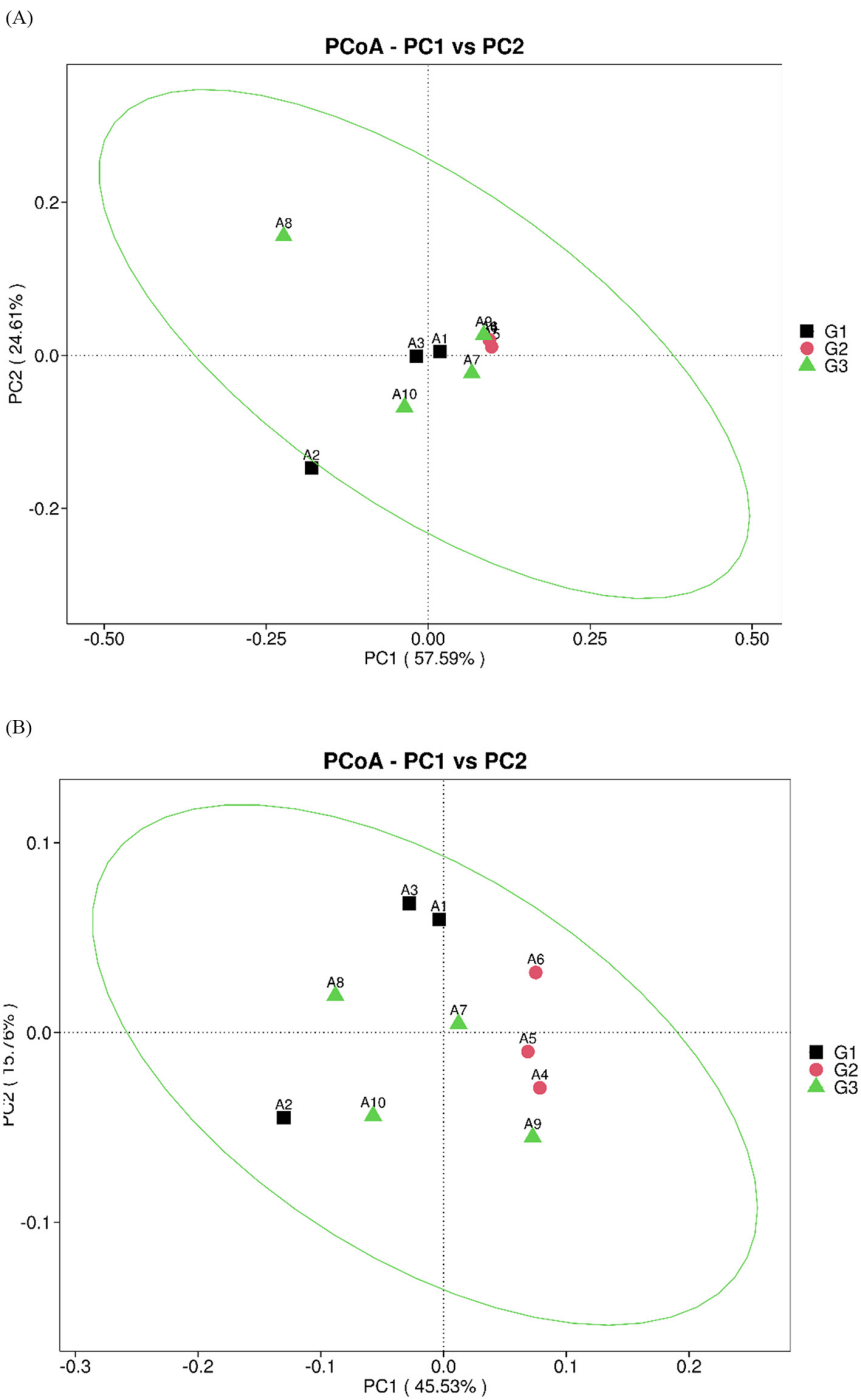
Dimension-reduction analysis based on species abundance. **(A)** Results of PCoA at the genus level. **(B)** Results of PCoA at the species level.

PCA based on the Bray-Curtis distance matrix revealed no significant differences in microbial composition at the genus and species levels among groups G1, G2, and G3 (Figures 2A, B).

### 3.3 Bacterial biomarkers at different physiological stages

**Figure 3:**
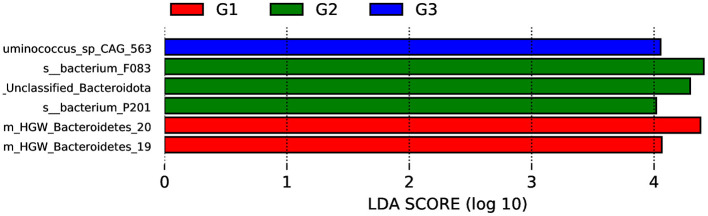
LDA score diagram for different species.

LEfSe analysis (LDA > 4, *P* < 0.05) was used to identify microorganisms with significant relative abundance variations in groups G1, G2, and G3. As shown in Figure 3, *Bacteroidetes* showed statistically significant differences between the groups. Next, we identified 12 biomarkers with statistically significant differences among the groups using the MetaGenomeSeq method, including *Acinetobacter_towneri, Empedobacter_falsenii, Comamonas_aquatica, Comamonas_kerstersii, Jeotgalibaca_porci, Pelistega_suis, Sarcina_ventriculi, Methanocorpusculum_labreanum, Candidatus_Methanarcanum_hacksteinii, Rothia_endophytica, Acinetobacter_baumannii, Aliarcobacter_cryaerophilus* (*P* < 0.05; Supplementary Data Sheet 1).

### 3.4 Functional characterization of gut microbiota

**Figure 4:**
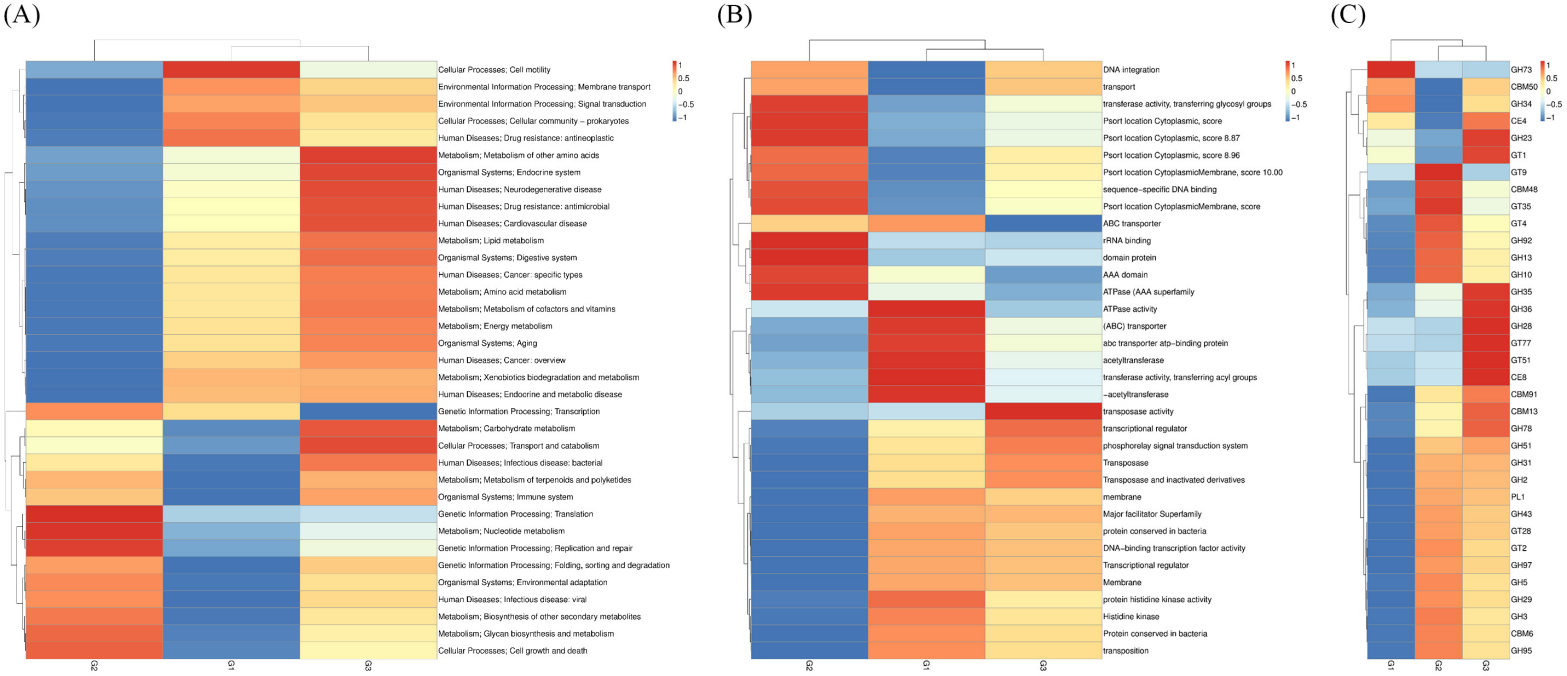
Functional characterization of gut microbiota in the G1, G2, and G3. **(A)** Distribution of relative abundances of KEGG pathway categories. **(B)** Distribution of relative abundances of eggNOG pathway categories. **(C)** Distribution of relative abundances of CAZy pathway categories.

An abundance clustering heatmap analysis was performed based on the functional annotations and abundance data of all samples within each database, focusing on functions that differed among the groups. Based on KEGG pathway, eggNOG, and CAZy comparisons, the gut microbiota in G1, G2, and G3 were enriched in various pathways (Figures 4A–C, Supplementary Data Sheets 4–6). Furthermore, based on abundance information, the KEGG orthologs with the highest relative abundance in G1 included “cell motility”, “membrane transport”, “signal transduction”, “cellular community”, and “drug resistance: antineoplastic” (Figure 4A, Supplementary Data Sheet 4); in G2, they included “translation”, “nucleotide metabolism”, and “replication and repair” (Figure 4B, Supplementary Data Sheet 5); and in G3, they included “metabolism of other amino acids”, “endocrine system,” and “neurodegenerative disease” (Figure 4C, Supplementary Data Sheet 6).

### 3.5 Fecal metabolites detected in Asian elephant based on LC-MS/MS

**Figure 5:**
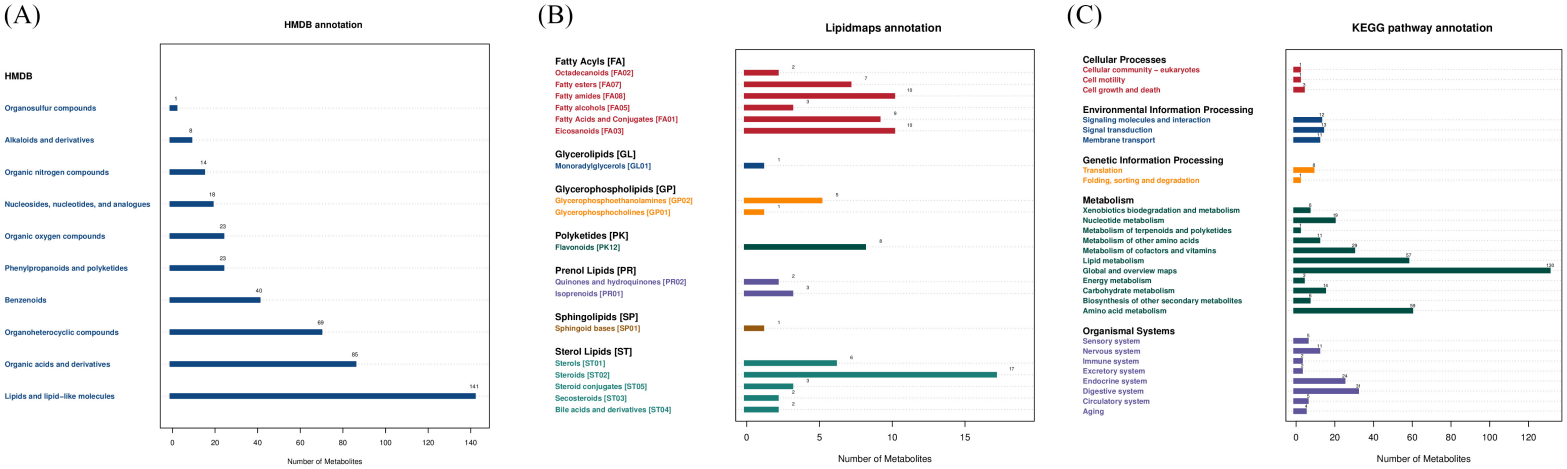
Annotation of all fecal metabolites detected in G1, G2, and G3. **(A)** HMDB classification of all metabolites. **(B)** LIPID MAPS annotation of all metabolites. **(C)** KEGG pathway annotation of all metabolites.

A total of 1,299 metabolites were identified across all samples by LC-MS/MS, with 857 and 442 metabolites detected in the positive and negative ion modes, respectively (Supplementary Data Sheet 7). HMDB annotation, LIPIDMAPS annotation, and KEGG pathway analysis showed that most differential metabolites were involved in lipid metabolism (Figures 5A–C, Supplementary Data Sheets 8–10).

### 3.6 Differential analysis and functional annotation of fecal metabolites

**Figure 6:**
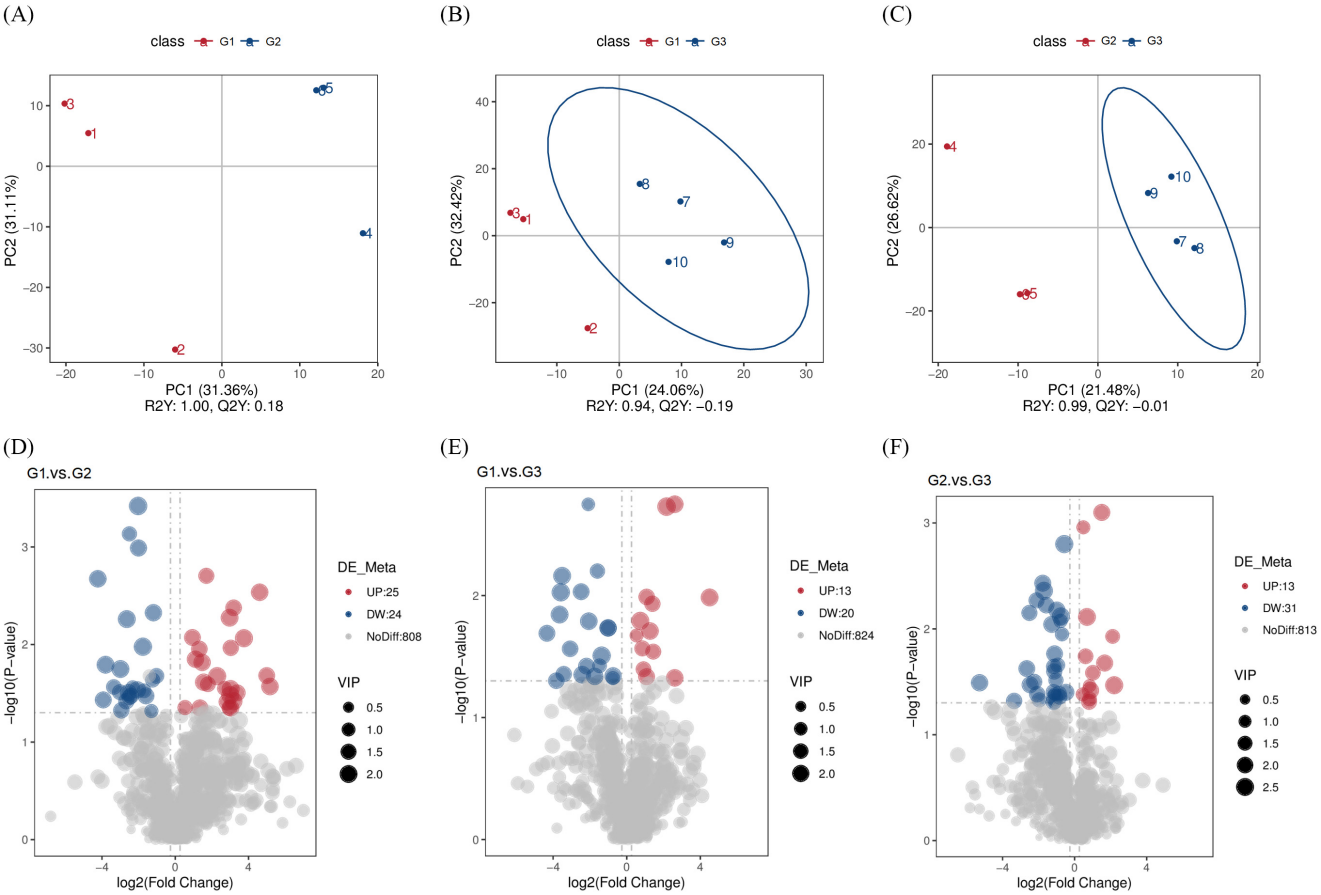
Differential analysis of fecal metabolites detected in groups G1, G2, and G3. **(A–C)** PLS-DA score scatter plots of differential metabolites in G1, G2, and G3. **(D–F)** Volcano plot of the differential metabolites in G1, G2, and G3.

Differential metabolites were identified using a supervised PLS-DA model with the following criteria: VIP > 1.0, fold change (FC) > 2 or FC < 0.5, and *P* values < 0.05 (*t*-test) (Figures 6A–C). The volcano plot of differential metabolites shows that there were 49 significantly altered metabolites between groups G1 and G2, among which 25 were upregulated and 24 were downregulated. Between G1 and G3, 33 significantly different metabolites were identified, including 13 upregulated and 20 downregulated. Comparing G2 and G3, 44 significantly different metabolites were detected, of which 13 were upregulated and 31 were downregulated (Figures 6D–F, Supplementary Data Sheet 11).

**Figure 7:**
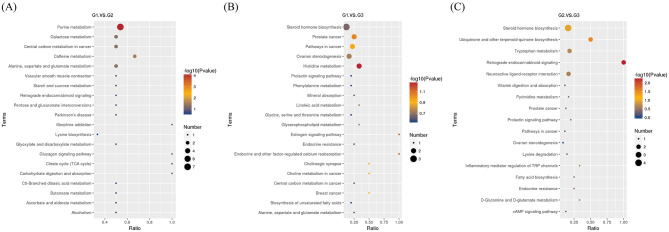
Bubble plots of the enriched KEGG pathways of differential metabolites. **(A)** Bubble plots of the enriched KEGG pathways of differential metabolites in G1 and G2. **(B)** Bubble plots of the enriched KEGG pathways of differential metabolites in G1 and G3. **(C)** Bubble plots of the enriched KEGG pathways of differential metabolites in G2 and G3.

KEGG pathway enrichment analysis was used to identify the main biological functions of the differentially expressed metabolites. Based on the enrichment results, a bubble chart showing the top 20 enriched KEGG pathways was generated. The results showed that “purine metabolism” (7 DMs) was annotated as the most differential metabolite pathway in G1 and G2, while “steroid hormone biosynthesis” (3 DMs and 4 DMs) was the most differential metabolite pathway in both G1 and G3, as well as in G2 and G3 (Figures 7A–C).

## 4 Discussion

The gut microbiota plays a significant role in reproductive regulation, including reproductive endocrinology and embryonic development, through interactions with hormones and other mechanisms (26–29). It affects the physiological functions of the host and is influenced by the living environment and feeding habits of the host animal (30). Therefore, a comprehensive understanding of the gut microbiota characteristics of captive Asian elephants, along with strategies to improve their intestinal health, is vital for enhancing fertility and protecting the population. This could provide a foundation for monitoring the health of the microbiota of captive animals and for age-specific dietary management. The gestational period of Asian elephants typically lasts for 22 months (31). During this period, the gut microbiota and metabolites of the mother may affect maternal health and fetal development (32, 33). Therefore, a comprehensive understanding of the different physiological stages of Asian elephants (growth, adulthood, and pregnancy) is important for their successful protection.

The results showed that there were differences in the bacterial content of the feces of the three groups of captive Asian elephants studied in this study; however, the differences among the three groups were not significant. The four bacterial phyla constitute the core components of the intestinal microbiota of mammals, including *Bacteroidota, Bacillota, Proteobacteria*, and *Actinobacteria* (34). Consistent with previous research results (35, 36), our study shows that the intestinal microbiota of the ten captive Asian elephants investigated in this study mainly consists of *Bacteroidetes* and *Bacillota*. The diversity of the gut microbiota is an important indicator of health and metabolic capacity (37). During pregnancy, mothers consume large amounts of food to provide essential nutrients for fetal growth and maternal health. Therefore, studies have found that the composition of the gut microbiota during pregnancy undergoes significant changes compared to that during non-pregnancy (9). In the G1 group and G2 group, there were some differences in the abundances of the *Bacteroidota* and *Pseudomonadota*, which were 16.53%, 26.60%, 11.50%, and 1.25%, respectively. However, there were differences in the composition of microorganisms among the G1, G2, and G3 groups through PCA and NMDS analyses, but these differences were not significant. This may be due to the captive environment and fixed diet (38). Further research is needed to better understand these findings.

In this study, *Bacteroidota* exhibited significant differences among groups. It was significantly increased in the G1 when compared to the other groups. *Bacteroidota*, the largest gram-negative bacterial phylum in the gastrointestinal tract, is considered a key contributor to the maintenance of health and complex homeostasis through the gut microbiota (39). Members of the *Bacteroidota* phylum appear to exhibit a high degree of metabolic flexibility and are capable of degrading dietary fiber (40). Females would consume a large amount of food during the pregnancy period. Dietary intakes in pregnancy were found to modify the *Bacteroidota* and positively influence the cell metabolism in pregnant women (41). At the genus level, *Acinetobacter, Lysinibacillus*, and *Solibacillus* exhibited the most significant differences. *Acinetobacter* was abundant in the G1 and G3 as compared to the G2 due to their body condition and high energy requirements (42). Non-pathogenic *Acinetobacter* may contribute to reproduction and are associated with host metabolism and health (43). It has been shown that the enrichment of *Acinetobacter* and the depletion of the potentially pathogenic bacteria were the most consistent changes in the gut microbiota of preterm infants supplemented with probiotics (44). A higher abundance of *Acinetobacter* sp. has been found positively related to a better physical condition in children (45). Although we observed mating, the individuals in Group G2 did not become pregnant after reaching sexual maturity, we are unsure whether their physical conditions were suitable for pregnancy. Furthermore, a decrease in *Acinetobacter* abundance may also lead to fetal developmental disorders (46). Some members of *Bacillus* are known to enhance reproductive performance and immunity in female livestock, thereby increasing offspring numbers (47–49). Therefore, compared to the other two groups, it is unsurprising that the abundance of *Bacillus* was lower in the G2.

Fecal metabolomics can be used to characterize the gut microbiota (50). Non-targeted metabolomic analysis provides a comprehensive approach for examining metabolic changes and identifying potential biomarkers of health and disease (51). Our metabolomic analysis of fecal samples revealed that the differences in metabolites among the groups were primarily concentrated in lipid metabolism. In addition to providing energy to the body, lipid metabolism plays a crucial role throughout the reproductive process (52). In this study, captive Asian elephants exhibited physiological changes in maternal lipid metabolism during pregnancy, which is essential for maintaining gestation and supporting fetal growth. Apart from glucose, triglycerides are the main source of energy for the fetus. Cholesterol plays a crucial role in the development of the embryo and fetus since it is part of cell membranes and lipid rafts (53). Fat breakdown and structure are affected by the gut microbiota, thereby enabling the production of lipoproteins in the intestines (54). Subsequently, maternal lipoproteins bind to specific receptors located on the syncytiotrophoblast cells of the placental villi (55). Therefore, lipid metabolism may be an important indicator for evaluating maternal health.

This study utilized the metagenomic and metabolomic data from the feces of 10 captive elephants to reveal the composition of their gut microbiota and metabolites. This provides important information on the composition and diversity of the gut microbiota in Asian elephants. Furthermore, although our sample includes subadult, pregnant adult, and non-pregnant adult elephants, the representativeness of the small sample size is extremely limited. Further research should expand the data collection scope to increase the number within each group and understanding the mechanism of the interaction among the microbiota in feces, metabolites, and host metabolism, as well as its role and significance in host health. In addition, the definition of groups was mainly based on the gestation period in this study. However, other factors might be more suitable when classifying based on the biological characteristics of elephants, a further study including the age range should be conducted.

In conclusion, our research provides baseline information on the gut microbiota communities of three pregnant, three never gotten pregnant after reaching sexual maturity, and three subadult captive Asian elephants. The further research results using multi-omics methods can provide information for more appropriate food management, which can promote the production of more beneficial bacteria in each elephant at the different physiological stages. In the future, it is necessary to study the differences in food management and actual nutritional components in order to promote the most effective management and nutrition for the healthy gut of captive elephants.

## Data Availability

The original contributions presented in the study are included in the article/Supplementary material, further inquiries can be directed to the corresponding author/s.
