# Supplementary material for: Gut microbiota and metabolic status during pregnancy in captive Asian elephants
Source: Front Vet Sci. 2026 Mar 9;13:1749490. doi: 10.3389/fvets.2026.1749490 (PMC13007507; doi:10.3389/fvets.2026.1749490)
Supplement: Supplementary file 1 [file Data_Sheet_1.docx]

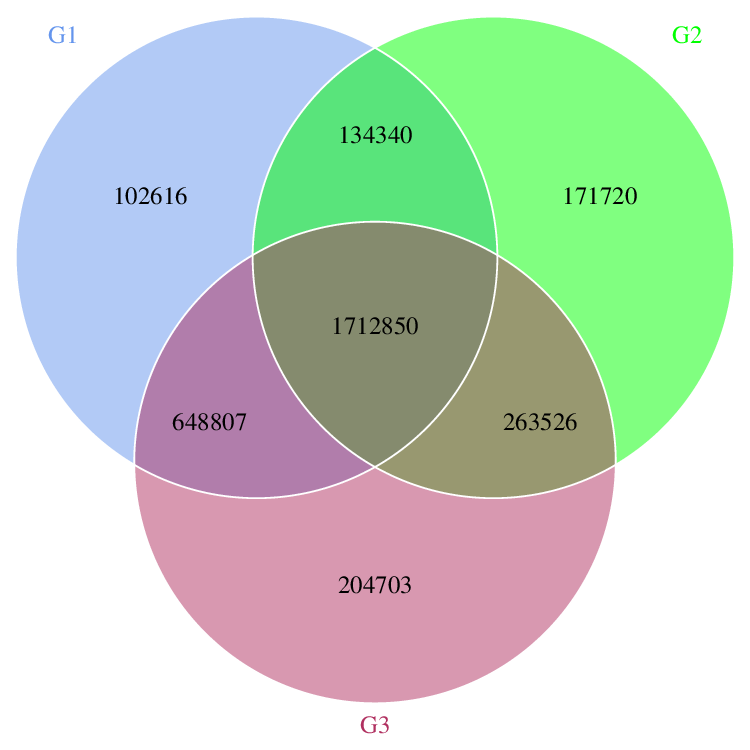


**Figure S1.** **Venn diagram of pregnant captive Asian elephants** (G1), non-pregnant captive Asian elephants (G2), and juvenile captive Asian elephants (G3). The numbers in the overlapping circles represent the number of genes shared among the groups; the numbers in the non-overlapping circles represent the number of genes unique to each group.

(A) (B)







(C) (D)







(E) (F)







(G) (H)







1. (J)







(K) (L)







**Figure S2.** **LEfSe and Metastat analysis of species differing among groups.** (A) Distribution map of LDA values for different species. The histogram displays species with LDA scores greater than 4, identifying biomarkers that show statistically significant differences between groups. The length of the bars represents the influence of each species. (A–L) Metastat analysis of gut microbiota at the species level. * indicates P value < 0.05, and ** indicates P value < 0.01.
